# Effects of an Adaptive Education Program on the Learning, Mental Health and Work Intentions of New Graduate Nurses

**DOI:** 10.3390/ijerph18115891

**Published:** 2021-05-31

**Authors:** Shu-Fen Chen, Yu-Wen Fang, Mei-Hua Wang, Tze-Fang Wang

**Affiliations:** 1 College of Nursing, National Yang Ming Chiao Tung University, Taipei 11221, Taiwan; moon2014100@gmail.com or; 2 Department of Nursing, Shuang Ho Hospital, Taipei Medical University, New Taipei City 23561, Taiwan; 3 Department of Nursing, Tzu Chi University of Science and Technology, Hualien City 970302, Taiwan; yvonnef1998@gmail.com; 4 School of Nursing, National Taipei University of Nursing and Health Sciences, Taipei 112303, Taiwan; meihua@ntunhs.edu.tw

**Keywords:** adaptive education program, new graduate nurses, transition, mental health, psychological distress, turnover rate

## Abstract

Health care workers are at a higher risk of psychological distress than ordinary people. Stress affects physical and mental health, and can even produce an intention to leave. The current training for new graduate nurses (NGNs) during this transitional period mostly focuses on the cultivation of professional ability, with less attention to mental health or emotional feelings, and thus there are insufficient structured support strategies. As such, this study explores the effects of intervention through an appropriate education program on the learning, mental health and work intentions of new recruits during the transition period. A pre-test and post-test for a single group was designed for new nursing staff in a large teaching hospital in northern Taiwan. The test period was from May 2017 to December 2018, and a total of 293 cases were accepted. A three-month adaptive education program was provided and evaluated in terms of: care for learning, care for health, improving professional ability, and individualized guidance on satisfaction, mental health disturbance and work intention. The new graduate nurses who received gentle care and counseling showed a downward trend in their BSRS-5 scores and statistical differences over time (*p* < 0.001). The higher the BSRS-5 score, the easier it is for new graduate nurses in acute and intensive care units. There is a tendency for turnover leave (*p* = 0.03). After the intervention of the overall plan, the turnover rate of new graduate nurses within three months was 12.6%, and the one-year retention rate was 87.9%. The adaptive education program uses multiple support strategies to improve learning and professional abilities, to reduce psychological emotions, and thereby to increase retention. Today will face new medical challenges; the education programs will become more important across clinical care settings, and it will be important to rigorously validate their performance in helping NGNs.

## 1. Introduction

The high turnover rate of nursing staff not only increases the depletion of hospital resources [1,2] but also the shortage caused by resignation will significantly affect the occurrence of healthcare and patient-adverse events [3,4]. Recent studies have shown that unhealthy lifestyles (poor sleep quality, depression, and conscious health impact, etc.) caused by busy work, high loads and high pressure will increase the intention of nursing staff to turnover and leave the job [5], and have found that especially the quality of sleep and depression have a great impact on the mental health and overall health of nursing staff [6,7].

According to the research, the risk of psychological symptoms for healthcare workers is much higher than that of general workers [8], and it is more likely to occur in nurses younger than 30 years old [9]. Psychological distress is an uncomfortable response or low mood caused by an individual’s response to specific stress or needs, and it can produce symptoms of depression and anxiety [10]. If the stressor cannot be effectively managed or responded to, it will be affect physical or mental health [11,12]. Studies have pointed out that medical and nursing students undergoing training are particularly prone to psychological distress such as anxiety and depression [7,13]. During the transition period, new graduate nurses (NGNs) switch from student status to being nursing staff, and are still in the learning stage for the working environment and professional role recognition [14]. Moreover, they face the challenge of acquiring the knowledge and skills required to become qualified professionals, and the variations in clinical care situations make the learning and adaptation at this stage extremely challenging [15]. Therefore, the highest physical or psychological stress will be presented during the first year of changing roles [16,17]. This is also a critical period that affects whether they will continue to stay in the nursing profession. The systematic literature suggests that it is necessary to develop appropriate screening measures for NGNs during the transition period, regularly monitor their fitness and physical and mental health, and provide diversified support strategies to increase retention [5,18,19].

Adaptive education is the application of different teaching measures to match the abilities, needs or interests of individual learners [20]. In order to provide such an education in a health professional field and develop an effective adaptive education plan, it is vital to prepare teachers in elements such as: respect for diversity, well-defined results, repeatability, the video recording of practice situations, and making sure all activities are trustworthy. The instructor and students need to operate in a safe environment [21].

For the enhancement of the professional abilities of new nurses, a trained preceptor plays an important professional role. A systematic literature review showed that a preceptor should have rich clinical care experience, effective teaching skills (listening, observation and feedback capabilities), and be able to role model in various capacities, such as: educator, consultant, assessor, facilitator (providing encouragement, support and improving skills) and other roles [22,23].

A learning style is a preference or strategy that learners adopt when facing learning and problems. It varies from person to person and is not good or bad, but is instead unique [24]. There are many types of scales used to measure learning styles, and the VARK learning styles model has often been mentioned in recent years. VARK is an abbreviation of the four main sensory methods, including visual, aural, read/write, kinesthetic and other different types [25]. Due to its simplicity, it is easy to understand, and allows learners to understand their own learning styles in order to enhance learning, and also allows clinical teachers to provide corresponding teaching strategies for participants’ different learning styles [26]. According to the research, clinical teachers should provide adaptive teaching based on the learning style of the new graduate nurses [27]. If the instructor can adjust the teaching method based on the learning style, they can improve the learning effect and reduce frustration [28]. In addition, the literature points out that the establishment of patient safety awareness and clinical practice by new nurses is still an important issue [14]. Objective structured clinical testing (OSCE) can effectively improve clinical performance [29,30]. New graduate nurses in the transitional period tend to lack confidence [31]; thus, if the clinical situation and OSCE can be combined, this will substantially improve their patient evaluation ability, professionalism and communication skills [32,33].

Based on the above, new graduate nurses need a holistic and programed intervention plan during the transition period to meet the needs of the transition period. Therefore, this research aims to design an appropriate diversified education program, and to explore the effects of the program on the learning, mental health and work intentions of new graduate nurses after the intervention.

## 2. Materials and Methods

### 2.1. Study Design and Participants

The pre-test and post-test design of a single group was targeted at new graduate nurses in a large teaching hospital in Taiwan. The conditions for the acceptance of a participant were new graduate nurses who had been employed within minimum three months of employment, and who had completed a five-day training course for new graduate nurses. The exclusion conditions were those who had worked outside the hospital. The initial number of participants was 384, although after applying the exclusion criterion, the sample was left with 293 participants from May 2017 to December 2018.

### 2.2. Intervention and Process

**Figure 1:**
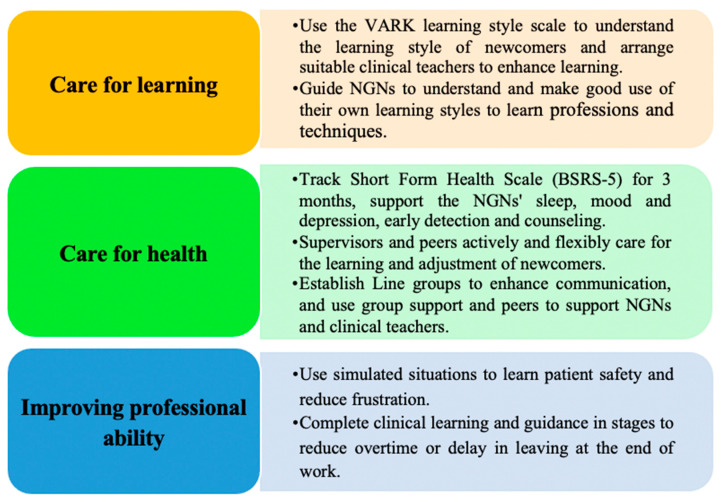
The three adaptive parts of the education program.

The adaptive education program of this study includes three parts: care for learning, care for health, and improving professional ability, as shown Figure 1. The duration was three months.

#### 2.2.1. Care for Learning

Starting from a learner-centered perspective, using the VARK learning style scale, new graduate nurses stated their preferred learning styles. Learning styles with more than one were classified as multiple styles. According to the results of the learning style of the new graduate nurses, the head nurse of the unit arranged a corresponding clinical teacher (preceptor) for guidance. Clinical teachers adjusted the teaching content or methods according to the learning style preferred by the new nurses, and provided guidance in different ways, such as by emphasizing visual or kinesthetic approaches.

The qualification of a preceptor is that they must be a full-time nurse with more than three years of clinical experience in the teaching hospital, and have at least 12 h of teaching ability training, have obtained teacher certification and have good interpersonal relationships. Their responsibilities are to act as educator, socializer, consultant, and role model for the new graduate nurses.

1. Educator: Regularly assess the learning needs of new graduate nurses, and design a learning plan that meets the needs on an individual basis. Help new recruits achieve their learning goals and often give positive feedback.
2. Socializer: Lead new recruits to understand the unit environment, work routines and resources. Take the initiative to support new recruits and make them feel welcome. Regularly meet with new graduate nurses or maintain close contact in order to understand the learning progress and difficulties of the new graduate nurses.
3. Consultant: Provide guidance on clinical case care for new graduate nurses, become the object of nursing consultation for new graduate nurses, and regularly participate in clinical teacher meetings.
4. Role model: With a wealth of knowledge, provide the correct and complete care of critical cases in the unit, and become the learning object for new graduate nurses.

#### 2.2.2. Care for Health

The preceptors and the unit nurses take the initiative to provide adjustment methods for the pressure of new graduate nurses:

1. Use the Short Form Health Scale (BSRS-5) to continuously track the physical and mental symptoms of the new graduate nurses for three months, supporting the sleep, mood and depression symptoms of newcomers, and offering early detection and counseling.
2. For those whose results of the Short Form Health Scale (BSRS-5) ≥ 10 points, the clinical teacher (preceptor) and the unit nursing chief provide active and gentle support and guidance, including life adaptation, work and study, interpersonal interaction, emotional adjustment, etc. In order to understand the psychological distress and pressure relief channels of new nursing staff, BSRS-5 results ≥ 15 points provide employees with free professional consultation channels, and it is recommended to consult a professional physician or psychologist for further evaluation.
3. Establish a line group to enhance multiple communication channels and peer support.

#### 2.2.3. Improve Professional Ability

**Figure 2:**
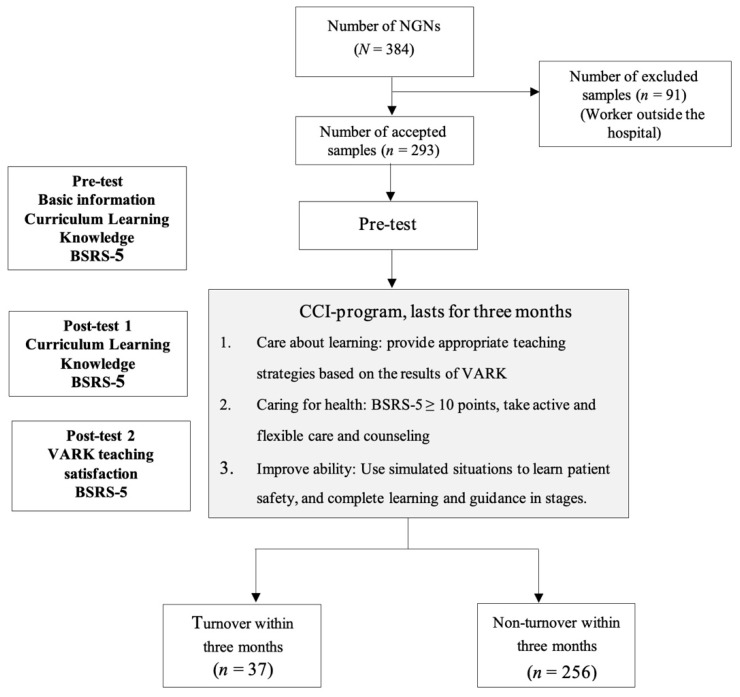
Flowchart of the study. BSRS-5: Brief Symptom Rating Scale.

Use phased goals to complete clinical guidance and learning:

1. The first part is five-day on-the-job training, which includes routines, standard operating procedures (SOP), common knowledge and common techniques, and evaluates the learning results by pre- and post-written tests, OSCE, DROPS and mini-CEX, etc.; learn about the safety of chemotherapy administration in a situational simulation mode in order to reduce frustration.
2. The second part is specialized training. The preceptors provide the learning basis and reference criteria for new graduate nurses based on the specialized attributes of the unit and combined with the clinical situation, and then provide immediate feedback or individualized reinforcement teaching based on the evaluation results in order to reduce frustration. The plan goals and content are adjusted according to the learning progress of the new graduate nurses, as shown Figure 2.

### 2.3. Study Tools

#### 2.3.1. Demographics

The basic information captured in this research includes the basic attributes of gender, education level, work unit and personal learning style. For the personal learning style, this study developed the VARK learning style scale after referring to the researcher’s questionnaire modification [24]. There are 16 questions on the scale, which are divided into visual learners, auditory learners, reading learners, and kinesthetic tactile learners. Accordingly, the new graduate nurses are asked to select their preferred learning styles, and learning styles with more than one type are classified as multiple styles.

#### 2.3.2. Instructor’s Individualized Teaching Satisfaction

The questionnaire was prepared by the researcher. It is mainly used to evaluate the satisfaction of the instructor after receiving the individualized guidance from the clinical teacher (preceptor) with the learner as the center. There are five questions in the questionnaire, each rated from 1~5 and scored with a Likert scale of 5 points, with 1 point meaning very dissatisfied and 5 points very satisfied.

#### 2.3.3. Psychological Distress

The Brief Symptom Rating Scale, BSRS-5 [34,35], was used to measure the current psychological distress of new graduate nurses during the transition period. There are five questions on the scale. The scoring method is based on the level of trouble, from 0 to 4 points: 0 means no trouble, 4 points means that the trouble or distress is very severe. For the total score of the first five questions, 0–5 points indicates good physical and mental adjustment, 6–9 points indicates mild emotional disturbance, 10–14 indicates moderate emotional disturbance, and 15 points or more indicate severe emotional disturbance. Cronbach’s alpha was 0.84, the sensitivity was 82.6%, and the specificity was 81.8% [34,35].

#### 2.3.4. Learning Effectiveness and Satisfaction

A self-developed professional knowledge questionnaire was used to formulate a score sheet for the learning content, with a total of 50 questions and a total score of 100 points. This questionnaire was tested according to a score sheet before the new graduate nurses accepted the course and after the course of 5 days. The Cronbach’s alpha of this study was 0.72. The nursing skills were evaluated by OSCE, including Intravenous administration, Intravenous injection, Urinary catheterization, Emergency equipment operation, and Defibrillator operation for a total of five technologies.

#### 2.3.5. Work Intention

The turnover rate within three months and the one-year retention rate were investigated:

1. Turnover rate within three months: the numerator is the number of new graduate nurses who resigned within 90 days of employment, and the denominator is the total number of new graduate nurses admitted in the study.
2. One-year retention rate: the number of new graduate nurses admitted in this study is the numerator, as well as the number of nurses who stayed in the hospital for more than 365 days. The denominator is the number of new graduate nurses who remained with the hospital for more than 365 days during the admission period of the study.

### 2.4. Statistical Analysis

This study used SPSS 20.0 software (IBM, Chicago, IL, USA) for the statistical analysis. A descriptive analysis was performed in order to calculate the means, standard deviations, numbers of participants, and percentages depending on the variables. In the advanced statistical analysis, a t-test analysis with independent groups and repeated measures ANOVA were conducted to determine the effectiveness of the counseling and care intervention, and the Generalized Linear Model was used to statistically analyze the unstructured model of the 3-month turnover rate, with *p* < 0.05 indicating significance.

## 3. Results

### 3.1. Demographic Details of the Participants

There were a total of 293 new nursing staff in this study, most of whom were female (91.8%), and 164 (56.0%) had a university education level or above. The number of people in general wards at work was the largest (61.4%). About half of the newly recruited nursing staff were auditory learners 151 (51.5%), followed by kinesthetic learners (23.5%), and a very small number (2%) were multiple types with more than one learning style (Multimodality) learners; the average score of BSRS-5 in the first month of employment was 5.39 ± 4.1 (Table 1).

### 3.2. Care for Learning

After the new graduate nurses received the individualized guidance of the clinical teacher (preceptor) centered on the learner, the average satisfaction rate for the 5-pointLikert scale was 4.61 ± 0.47 (Table 2). As analyzed by the learning style, the average satisfaction of visual learners was 4.74 ± 0.46, followed by the satisfaction of multimodality learners at 4.72 ± 0.44, but for learners with different learning styles there was no statistical difference in satisfaction (*p* < 0.35).

### 3.3. Care for Health

**Figure 3:**
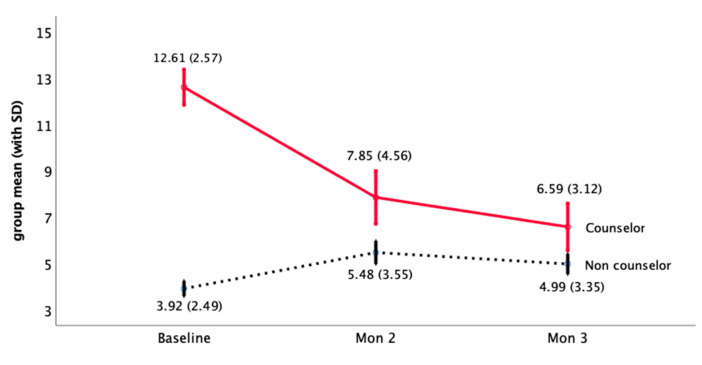
Mental health distress score trend of NGNs receiving gentle care counseling.

When the new graduate nurses had a BSRS-5 ≥ 10 points, the clinical teacher (preceptor) and the unit nursing chief proactively provided gentle care and guidance. In this study, 41 new graduate nurses received gentle care counseling, and the average score of the BSRS-5 decreased from 12.61 ± 2.57 to 6.59 ± 3.12 over time (Figure 3). For those who did not receive gentle care counseling, the BSRS-5 score increased slightly from 3.92 ± 2.49 to 4.99 ± 3.35. The statistical results show that there are statistical differences between the effects within-subjects (time or the interaction between group and time) and between-subjects (group) (*p* < 0.001), showing that not receiving gentle care counseling compared with those who received gentle care and counseling, the BSRS-5 of new graduate nurses has a downward trend and reaches a statistical difference (Table 3).

### 3.4. Improving Professional Ability

Most of the new graduate nurses improved their average scores of professional knowledge after the class (Table 4). Among them, those who received gentle care coaching (10.54 ± 7.82) had more progress in professional knowledge than those who did not receive gentle care coaching (7.57 ± 7.63), and this reached a statistical difference (t = 2.27, *p* = 0.02).

### 3.5. The Turnover Tendency of New Graduate Nurses within Three Months

According to the statistics, 37 (12.6%) of new graduate nurses that turnover leave their jobs within 90 days of employment. According to the analysis of a Generalized Linear Model (Table 5), after controlling for disturbing factors such as education level, personal learning style, satisfaction with the preceptor, and professional knowledge scores compared with the general ward, the new graduate nurses in the emergency and intensive care unit with a higher BSRS-5 for the first time on the job are more likely to have the tendency to resign, and this is statistically significant (*p* = 0.03).

## 4. Discussion

The adaptive education program of this research provides a structured support strategy for new nurses during the transition period, through the intervention of the three major aspects of a multi-strategy of care that addresses the learning of new graduate nurses, physical and mental health, and the enhancement of professional ability. Care and support effectively increased the one-year retention rate of new graduate nurses during the transition period. The results of the study are similar to those mentioned in a systematic review regarding the ways in which a support strategy with a structured design can influence new graduate nurses’ willingness to remain in the profession [18].

In addition, this research aimed to support new nurses with negative mental health problems, providing feedback, encouraging positive ideas and interactions for 4 weeks’ time, and through continuous tracking and monthly evaluation, showing that positive improvement results can be achieved. This result is similar to the results in the literature that use mindfulness-based interventions to reduce emotional distress [36]. It is also similar to use of the Mindbodystrong Program for 8 weeks—45 min a week—to improve mental health and other effects [37]. However, the pressure of different clinical working environments may affect the quality of patient care and potential patient safety issues [38]. The frustrations and demands derived from clinical learning are one of the sources of work pressure for new nurses. The literature points out that the development of emotional intelligence is a useful auxiliary means to improve clinical performance and reduce the risk of clinical emotional distress [39]. The global outbreak of coronavirus disease (COVID-19) began in December 2019 and has lasted until now. The high levels of stress experienced by nurses during this pandemic may have immediate and long-term effects on their mental health [40]. In addition to considering the characteristics of each medical environment, it is necessary to formulate specific plans after focusing on the learning characteristics and psychological feelings of the personnel. The preliminary results of this research are expected to provide a specific reference direction.

This study shows positive results for the work intention of new graduate nurses with a one-year retention rate of 87.9%. The effectiveness of the study intervention is higher than the average one-year retention rate of 75% according to the Asian survey data [41] and the analysis of US data showing an average one-year retention rate of 83% [42]. There are two possible reasons for the effectiveness of the flexible care intervention in this study. One is the regular assessment of needs and the adjustment plan. This is the same as research results indicating that the stressors of new graduate nurses of different generations will change according to the clinical situation at the time [43]. Secondly, the intervention objects of this study also included the care of the instructor, which is related to the results of a literature review pointing out that a positive preceptor experience and self-confidence in a supportive environment are keys to retaining new graduate nurses [44]. Researchers have pointed out that relevant intervention measures such as respect, autonomy, and quality of work life, etc., can increase the retention rate of those with an intention to leave [45]. However, some literature results pointed out that although similar nurse residency programs (NRPs) are used, the 1-year retention rate for new graduate nurses varies from 74% to 100% [46]. In summary, managers must adjust the intervention plan in a timely manner based on the actual working conditions of the new nurses, and more studies are needed to confirm the effectiveness of the intervention.

This study provided adaptive teaching based on the learning style of new graduate nurses, and auditory learners were at most more than half, followed by tactile learners (kinesthetic). The results of this study are similar to other studies indicating that choosing a teaching method that matches the learning style of the students increases participation and satisfaction [47,48], and also improves nursing staff anxiety and stress [49]. In addition, the results of this study show that different learning styles do not affect work orientation. This result is similar to a randomized controlled study pointing out that the matched learning style teaching method can reduce the number of wrong attempts, but it is not statistically significant [50]. However, the learning style is a dynamic change [27], and different countries or education systems may have different learning style preferences [51]. For example, Australian nursing masters degree graduate students tend to be kinaesthetic [52]. Based on the above, we must consider the diversity of learners when designing teaching programs.

This study has several limitations. First, this study was only conducted in a teaching hospital and included every qualified person, and was not a randomized controlled design, such that the extrapolation of the results is limited. Second, the gentle care plan for the individuals in this study will be affected by personal acceptance, so suitability must be considered when applying this plan. Third, the study may have possible bias when using questionnaires and scales. Fourth, this study was designed for the retention rate and the needs of the job adaptation of NGNs. Therefore, the impact of burn-out was not involved. Finally, the case severity for the clinical care varied in each work unit during the period of this study, which may affect the applicability of the research results. Therefore, the application of the results of this study must be carefully considered and evaluated.

## 5. Conclusions

This study designed a structured and adaptive learning program that can improve the emotions of new nursing personnel and enhance their work intention. The present results can be used as a reference for supervisors in similar hospital environments when formulating strategies. In today’s dynamic healthcare environment, the nurse manager should also seek ways to integrate the process that the NGNs progress through during the first year of practice, as well as throughout the new nurse’s career, with his/her performance expectations and develop more professional education resilience training courses to foster learning and adaptability. Under the current impact of the COVID-19 epidemic, nursing staff’s physical and mental pressure has increased, and it is necessary to increase the stress adjustment and stress-relief methods. Therefore, exploring nurses’ stress and mental problems during this pandemic and applying psychological support combined with this research will be the target direction in the future.

## Figures and Tables

**Table 1:** Demographics and clinical characteristics (*N* = 293).

Participants’ Characteristics	*n*	%
Sex		
Female	269	91.8%
Male	24	8.2%
Education		
<Bachelor’s degree	129	44.0%
≥Bachelor’s degree	164	56.0%
Department		
General Wards	180	61.4%
Critical care unit ^1^	58	19.8%
Special units ^2^	55	18.8%
Learning style		
Visual	45	15.4%
Auditory	151	51.5%
Reading/Writing	22	7.5%
Kinesthetic	69	23.5%
Multimodality	6	2.0%
BSRS-5 ^3^ (mean, SD)	5.39	4.10

^1^ Critical care unit: emergency room, intensive care unit; ^2^ Special units: others; ^3^ BSRS-5: Brief Symptom Rating Scale results in the first month of employment.

**Table 2:** Satisfaction of NGNs with different learning styles receiving preceptor-individualized teaching.

		Mean	S.D.	95% CI		F	*p*
	*n*			Lower	Upper		
Visual	45	4.74	0.46	4.60	4.88	1.11	0.35
Auditory	151	4.58	0.46	4.50	4.65		
Reading	22	4.56	0.55	4.31	4.82		
Kinesthetic	69	4.61	0.46	4.49	4.72		
Multimodality	6	4.72	0.44	4.18	5.26		
Total	293	4.61	0.47	4.56	4.67		

**Table 3:** Analysis of the mental health distress of NGNs receiving gentle care and counseling.

		1st Month (Baseline) BSRS-5		2nd Month BSRS-5		3rd Month BSRS-5		Within-Subjects		Between-Subjects
	*n*	Mean	SD	Mean	SD	Mean	SD	Time	Time × Group	Group
Non counselor	215	3.92	2.49	5.48	3.55	4.99	3.35	36.75	87.91	95.51
Counselor	41	12.61	2.57	7.85	4.56	6.59	3.12	<0.001	<0.001	<0.001
Total	256	5.31	4.06	5.86	3.82	5.24	3.36			

× interaction.

**Table 4:** Variate analysis of the professional knowledge scores for NGNs receiving gentle care and counseling.

		Pre-test		Post-test		Variation			
	*n*	Mean	SD	Mean	SD	Mean	SD	t	*p*
No Counselor	215	80.89	8.79	88.50	8.07	7.57	7.63	2.27	0.02
Counselor	41	77.85	6.28	88.39	7.32	10.54	7.82		

**Table 5:** GLM analysis of the NGN turnover leave within 90 days.

Parameter	B	S.E.	*t*	*p*	95% CI	
					Lower	Upper
Intercept	0.17	0.32	0.55	0.59	−0.46	0.80
Education ^1^						
<Bachelor’s degree	0.02	0.04	0.55	0.58	−0.06	0.10
Learning style ^2^						
Visual	0.15	0.15	1.00	0.32	−0.15	0.45
Auditory	0.12	0.15	0.83	0.40	−0.17	0.41
Reading	0.16	0.16	1.02	0.31	−0.15	0.48
Kinesthetic	0.11	0.15	0.73	0.47	−0.19	0.40
Satisfaction with Preceptor	−0.07	0.04	−1.65	0.10	−0.15	0.01
Pre-test for Professional Knowledge Questionnaire	0.001	0.003	0.43	0.67	−0.004	0.001
Post-test for Professional Knowledge Questionnaire	0.001	0.003	0.24	0.81	−0.015	0.001
BSRS-5 (Baseline) × Special units ^3^	−0.01	0.01	−0.70	0.48	−0.04	0.02
BSRS-5 (Baseline) × Critical care unit ^3^	0.03	0.01	2.20	0.03	0.003	0.05

Reference group: ^1^ ≥ bachelor’s degree, ^2^ multimodality, ^3^ BSRS-5 (baseline) × wards; × interaction.

## Data Availability

The data that support the findings of this study are available from the first author.
